# Supplementary material for: Current Opportunities for Targeting Dysregulated Neurodevelopmental Signaling Pathways in Glioblastoma
Source: Cells. 2022 Aug 15;11(16):2530. doi: 10.3390/cells11162530 (PMC9406959; doi:10.3390/cells11162530)
Supplement: Supplementary file 1 [file cells-11-02530-s001.zip › cells-1783232-supplementary.pdf]

Supplementary Table S1. Stratification of GBM into molecular subtypes and characteristics of subtypes.

Stratification	Subtypes	Characteristics of the subtype
**Based on IDH1/2 mutations**	*IDH wild-type GBM*	TERT promoter, PTEN, TP53, PIK3CA, PIK3R1, NF1, H3F3A mutation
		EGFR, PDGFRA, MET, CDK4, CDK6, MDM2, MDM4 amplification
		EGFRvIII deletion
		MGMT promoter methylation
		trisomy 7, 7q gain, monosomy 10, double minute chromosomes
	IDH mutant GBM	IDH1 or IDH2, TP53, ATRX mutation
		homozyguous CDKN2A/p14^{ARF} deletion
		MGMT promoter methylation
		trisomy 7 or 7q gain, LOH 17p, 10q deletion
**Based on the 2016 WHO classification and cIMPACT- NOW recommendations**	*GBM*	no mutations in *IDH* genes
		No mutations in histone *H3* genes
		microvascular proliferation, necrosis and/or molecular features, including mutation in *TERT* promoter, *EGFR* gene amplification and/or +7/–10 cytogenetic signature
	IDH- mutant astrocytoma, WHO grade 4	homozygous deletion of *CDKN2A/B* locus
		mutations in *IDH* genes
		loss of nuclear *ATRX*
**Based on gene expression profiles**	*Proneural*	*PDGFRA* alterations
		point mutations in *IDH1*
		TP53 mutations and loss of heterozygosity
		chromosome 7 amplification paired with chromosome 10 loss
		high expression of oligodendrocytic development genes such as *PDGFRA*, *NKX2-2* and *OLIG2*
		PIK3CA/PIK3R1 mutations
		expression of proneural development genes such as *SOX* genes, *DCX*, *DLL3*, *ASCL1* and *TCF4*
	Classical	EGFR amplification
		point or vIII EGFR mutation
		lack of TP53 mutations
		CDKN2A homozygous deletion
		chromosome 7 amplification paired with chromosome 10 loss
		affected RB pathway

		high expression of *NES*
		high expression of the Notch and SHH pathways
	Mesenchymal	*NF1*, *PTEN* mutations
		hemizygous deletions of a region at 17q11.2, containing the *NF1* gene
		high frequency of *NF1* mutation/deletion
		high expression of mesenchymal markers (*CHI3L1*, *MET*)
		increased NF-kB pathway
	Neural	expression of neuronal markers (such as *NEFL*, *GABRA1*, *SYT1*, *SLC12A5*)
**Based on gene expression and DNA methylation profiles**	*Proneural*	upregulation of genes enriched in cell division pathways, downregulation of genes enriched in inflammatory pathways
	Classical	upregulation of genes of ErbB receptor tyrosine kinase family signaling pathway and cell fate commitment, downregulation of genes of immune system-related pathways
	Mesenchymal	higher expression of genes in the ERK1/2 cascade, activation of pathways associated with immunity and inflammation processes, downregulation of DNA repair genes and genes enriched in RNA splicing pathways
**Based on integrative approach combining scRNA-seq, TCGA deconvolution and experimental models**	*NPC-like 1*	expression of *SOX4*, *DCX*, *CD24*, *DLL3*, *SOX11*
	NPC-like 2	expression of *RND3*, *SOX11*, *DCX*, *CD24*, *STMN4*, *STMN2*, *DLX5*, *DLX6-AS1*
	OPC-like	expression of *PLP1*, *ALCAM*, *OLIG1*, *OMG*, *PLLP*
	AC-like	expression of *CST3*, *GFAP*, *S100B*, *HOPX*, *SLC1A3*, *MLC1*
	MES-like 1	expression of *VIM*, *ANXA1*, *ANXA2*, *CHI3L1*, *CD44*
	MES-like 2	expression of *DDIT3*, *ENO2*, *VIM*, *ADM*, *LDHA*, *HILPDA*
**Cell lineage-based stratification**	*type I*	expression of *EGFR* and *SOX9*
		expression of *SLC1A3* gene
		enrichment of biological processes that reflect astrocyte differentiation and cell migration
	type II	Expression of *ERBB3* and *SOX10*
		enrichment of biological processes that reflect the myelination, gliogenesis, and oligodendrocyte differentiation
		increased amplification of genes in the vicinity of PDGFRA and KIT locus

		expression of *FA2H* gene
**Based on results of single-cell RNA sequencing**	*developmental state*	high proliferation
		expression of genes encoding products associated with neurodevelopmental programs
	injury response state	mesenchymal-like gene-expression signatures
		expression of genes encoding products indicative of hypoxia, reactive astrocytes, and wound-healing response
**Based on immunohistochemical analysis, DNA copy number and DNA methylation profiles**	*OPC type*	expression of PDGFRA, p16, p53 and OLIG2
		IDH1 and TP53 mutations
		methylation of the MGMT promoter
	DOC type	OLIG2 expression
		1p/19q co-deletion
		CDKN2A loss
	AsMes type	strong expressions of nestin, CD44, GFAP and podoplanin
		low levels of Olig2, IDH1-R132H, p53, p16, PDGFRA
	Mixed type	frequent expressions of p16, EGFR, Hes-1, p53
		Activation of Notch-Hes-1 pathway
		EGFR amplification
		low frequency of CDKN2A loss
		Expression of CD44 and GFAP
**Based on growth factors and cytokines expression profiles**	*type I*	high expression of TAC1, CCK, CHGA, PNOC, SST, FGF17, CHGB, and NPY
		enriched for the genes down-regulated by KRAS signaling
	type II	high expression of SAA1/2, RETN, CXCL8
		enriched in gene sets of epithelial-mesenchymal transition, KRAS signaling up, mTORC1 signaling, P53 pathway, and PI3K/AKT/mTOR signaling
	type III	high expression of PDGFRA, ESM1, CS
		enrichment in the two cell cycle-related gene sets (E2F targets and G2M checkpoint PG5)

This summary is based on the previously reported publications listed in the Manuscript, subsection Molecular subtypes of glioblastoma. TERT - telomerase reverse transcriptase; PTEN - phosphatase and tensin homolog on chromosome 10; TP53 - tumor protein 53; PIK3CA - phosphatidylinositol 3-kinase, catalytic, alpha; PIK3R1 - phosphatidylinositol 3-kinase regulatory subunit 1; NF1 – neurofibromatosis type 1; H3F3A - histone H3 family 3A; EGFR – epidermal growth factor receptor; PDGFRA - platelet-derived growth factor receptor A; MET - hepatocyte growth factor receptor; CDK4 - cyclin dependent kinase 4; CDK6 - cyclin dependent kinase 6; MDM2 – mouse double minute 2; MDM4 - mouse double minute 4; EGFRvIII -

epidermal growth factor receptor variant III; MGMT - O6-methylguanine-DNA methyltransferase; IDH1 - isocitrate dehydrogenase 1; IDH2 - isocitrate dehydrogenase 2; ATRX – nuclear alpha-thalassemia/mental retardation syndrome, X-linked; CDKN2A - cyclin-dependent kinase inhibitor 2A; NKX2-2 - NK2 Homeobox 2; OLIG2 - Oligodendrocyte Transcription Factor 2; PIK3CA - phosphatidylinositol-4,5-bisphosphate 3-Kinase catalytic subunit alpha; PIK3R1 - Phosphoinositide-3-Kinase Regulatory Subunit 1; SOX - Sry-related HMG box; DCX – doublecortin; DLL3 - Delta Like Canonical Notch Ligand 3; ASCL1 - Achaete-Scute Family BHLH Transcription Factor 1; TCF4 - transcription Factor 4; NES – nestin; CHI3L1 - Chitinase 3 Like 1; NEFL - neurofilament light chain; GABRA1 - Gamma-Aminobutyric Acid Type A Receptor Subunit Alpha1; SYT1 - synaptotagmin 1; SLC12A5 - solute carrier family 12 member 5; RND3 - Rho Family GTPase 3; ERBB3 - ErbB receptor tyrosine kinase 3; SLC1A3 - solute carrier family 1 member 3; KIT - KIT Proto-Oncogene, Receptor Tyrosine Kinase; FA2H - Fatty Acid 2-Hydroxylase; STMN4 - Stathmin 4; STMN2 - Stathmin 2; DLX5 - Distal-Less Homeobox 5; DLX6-AS1 - DLX6 Antisense RNA 1; PLP1 - Proteolipid Protein 1; ALCAM - Activated Leukocyte Cell Adhesion Molecule; OLIG1 - oligodendrocyte transcription factor 1; OMG - Oligodendrocyte Myelin Glycoprotein; PLLP – Plasmolipin; CST3 - Cystatin C; GFAP - glial fibrillary acidic protein; S100B - S100 Calcium Binding Protein B; HOPX - HOP Homeobox; MLC1 - Modulator Of VRAC Current 1; VIM – vimentin; ANXA1 - Annexin A1; ANXA2 - Annexin A2; DDIT3 - DNA Damage Inducible Transcript 3; ENO2 - Enolase 2; ADM – Adrenomedullin; LDHA - Lactate Dehydrogenase A; HILPDA - Hypoxia Inducible Lipid Droplet Associated; Hes-1 - Hes Family BHLH Transcription Factor 1; TAC1 - tachykinin precursor 1; CCK – Cholecystokinin; CHGA - Chromogranin A; PNOC – prepronociceptin; SST – somatostatin; FGF17 - fibroblast growth factor 17; CHGB - chromogranin B; NPY - neuropeptide Y; SAA1/2 - serum amyloid A1/2; RETN – resistin; CXCL8 - C-X-C Motif Chemokine Ligand 8; ESM1 - endothelial cell specific molecule 1; CS - citrate synthase.
